# Differential diagnostic value of plain CT scan in adrenal adenoma and non-adenoma: A two-center control study of mean attenuation value, minimum attenuation value, and CT histogram

**DOI:** 10.3389/fendo.2022.1007870

**Published:** 2022-11-09

**Authors:** Zhijiang Han, Mengwei Wu, Peiying Wei, Hanlin Zhu, Xiaohan Zhang, Zhongxiang Ding, Ming Zhang

**Affiliations:** ^1^ Department of Medical Imaging, The First Affiliated Hospital of Xi’an Jiaotong University, Xi’an, China; ^2^ Department of Radiology, Affiliated Hangzhou First People’s Hospital, Zhejiang University School of Medicine, Hangzhou, China; ^3^ Department of Radiology, The Quzhou Affiliated Hospital of Wenzhou Medical University, Quzhou People’s Hospital, Quzhou, China; ^4^ Department of Radiology, Hangzhou Ninth People’s Hospital, Hangzhou, China; ^5^ Department of Radiology, Zhejiang Xiaoshan Hospital, Hangzhou, China; ^6^ Department of Radiology, Key Laboratory of Clinical Cancer Pharmacology and Toxicology Research of Zhejiang Province, Affiliated Hangzhou First People’s Hospital, Zhejiang University School of Medicine, Hangzhou, China

**Keywords:** adrenal gland neoplasms, adrenal adenoma, computed tomography, attenuation value, CT histogram

## Abstract

**Objectives:**

To investigate the value of mean attenuation value (AV_mean_), minimum attenuation value (AV_min_), and CT histogram (CTH) for the differential diagnosis of adrenal adenoma and non-adenoma in two medical centers.

**Methods:**

The plain CT data of 403 cases of adrenal adenoma and 141 cases of non-adenoma in center A were retrospectively analyzed, and compared with data of 86 cases of adenoma and 71 cases of non-adenoma in center B. All cases were confirmed by pathology or clinical follow-up. The diagnostic efficacy of AV_mean_ ≤ 10 Hounsfield units (HU), AV_min_ ≤ 0 HU, and CTH negative pixels ≥ 10% for adrenal adenoma, and AV_min_ and CTH for adenoma with AV_mean_ > 10Hu were compared between the two medical centers.

**Results:**

In medical centers A and B, the AUC of AV_mean_ for the differential diagnosis of adenoma and non-adenoma was 0.956 and 0.956, respectively, and the corresponding sensitivity, specificity, and accuracy were, 0.591 and 0.663, 1.000 and 1.000, 0.697, and 0.815, respectively, when the threshold was ≤ 10 HU. The AUC of AV_min_ was 0.941 and 0.958, respectively, and the corresponding sensitivity, specificity, and accuracy were 0.869 and 0.826, 0.986, and 0.972, 0.899, and 0.892, respectively, when the threshold was ≤ 0 HU. The AUC of CTH negative pixels was 0.948 and 0.952, respectively, and the corresponding sensitivity, specificity, and accuracy were 0.759 and 0.674, 1.000 and 1.000, 0.822, and 0.822, respectively, when the threshold was ≥ 10%. Among adenoma with AV_mean_ >10 HU, the best threshold of AV_min_ in center A and center B were -0.250HU and 2.375HU, and the corresponding AUC, sensitivity and specificity were 0.858 and 0.846, 0.691 and 0.586, 0.986 and 0.958; the best threshold of CTH in center A and center B were 0.895% and 0.775%, and the corresponding AUC, sensitivity and specificity were 0.873 and 0.822, 0.818 and 0.724, 0.837 and 0.915.

**Conclusion:**

AV_mean_, AV_min_, and CTH are all important parameters for differentiating adrenal adenoma from non-adenoma. Even for adenomas with AV_mean_ > 10 HU, AV_min_ and CTH still had high diagnostic efficiency. The three parameters are complementary, assisting clinicians to develop personalized treatments.

## Introduction

The detection rate of adrenal incidentalomas gradually increases with the application of cross-sectional imaging, accounting for approximately 4-10% of all abdominal computed tomography (CT) examinations (1–4). Adrenal incidentalomas include solid tumors, such as adrenal adenoma, adrenal pheochromocytoma, adrenocortical carcinoma, and adrenal metastasis, of which adrenal adenoma accounts for 70-80% (4, 5). Most adrenal adenomas do not have endocrine functions and only need to clinical follow-up (1, 2). However, adrenal pheochromocytomas mainly secrete catecholamines, leading to the elevated blood pressure, and adrenal cortical carcinomas can invade surrounding tissues and distant metastasis, while adrenal metastasis involves precise clinical staging of the primary tumors, all of which require early clinical intervention. Therefore, it is extremely important to clarify the nature of adrenal incidentalomas to develop personalized treatments.

Plain CT, adrenal washout CT, and chemical shift magnetic resonance imaging (MRI) are important imaging methods to identify adrenal incidentalomas, of which plain CT is a simple, economic, and more commonly used method. In plain CT, adrenal adenomas mainly have a low-density due to different proportions of intracellular lipids. Therefore, the amount of lipid components in tumors can be reflected by mean attenuation value (AV_mean_), minimum attenuation value (AV_min_), and CT histogram (CTH), and an adenoma can be therefore diagnosed. To our knowledge, AV_mean_ is the most commonly used parameter for the diagnosis of adrenal adenoma. At present, the widely accepted threshold value for AV_mean_ is ≤ 10 Hounsfield units (HU). Although its specificity of the diagnosis of adenoma can reach 93.3-100%, its sensitivity is somewhat insufficient and varies greatly, ranging from 47.6% to 82.2% (6–10). CTH is another method to diagnose adrenal adenoma. The widely accepted threshold for CTH is negative pixels ≥ 10%, which is similar to the specificity of AV_mean_ ≤ 10 Hu, and its sensitivity is improved to some extent, ranging from 82.9% to 92.2% (7–9). However, the acquisition of CTH is complicated and requires a special post-processing workstation, which is difficult to be popularized and applied in imaging diagnostic terminals. In 2015, we, for the first time, used AV_mean_ ≤ 0 HU in the differential diagnosis of adrenal adenoma and non-adenoma (11), and the results showed that the sensitivity and accuracy of AV_mean_ ≤ 0 HU were significantly higher than those of AV_mean_ ≤ 10 HU, and the specificity was slightly lower than that of AV_mean_ ≤ 10 HU. It is noteworthy that if AV_mean_, CTH, and AV_min_ can be combined to achieve complementary advantages, it may improve the differential diagnosis of adrenal adenoma by CT plain to a certain extent, avoid unnecessary radiation exposure and adverse reactions of contrast agents, and reduce the patient’s visiting time and medical expenses.

In the present study, AV_mean_, AV_min_, and CTH were used to conduct a comparative study of adrenal adenoma and non-adenoma in two medical centers based on plain CT data, in order to explore the diagnostic efficacy and stability of these three parameters, so as to provide an important basis for clinical treatment decisions.

## Methods

### Study population

**Figure 1:**
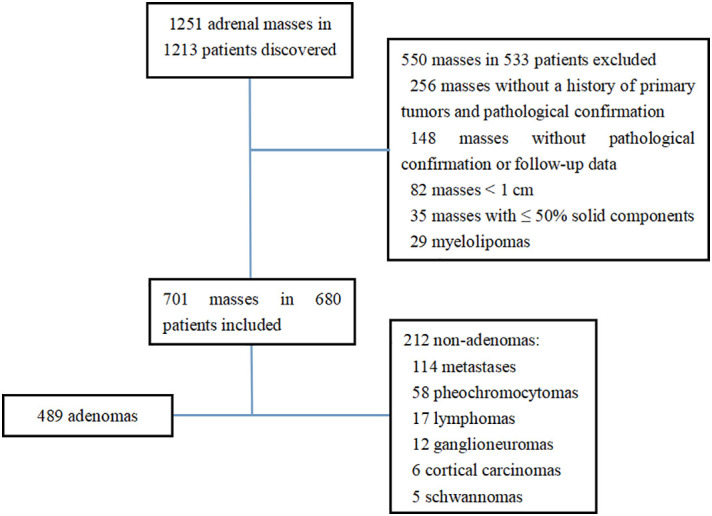
Flowchart of study enrollment.

This study was performed in accordance with the ethical standards of the two institutions. Due to the retrospective design of the study, informed consent from the patients was waived according to national policy. The plain CT data of adrenal tumors in medical centers A and B from January 2012 to December 2021 were retrospectively analyzed. Initially, 1251 adrenal masses in 1213 patients were found in the two medical centers. The inclusion criteria were as follows: 1) patients who were confirmed by pathology or clinical follow-up; 2) the minimum diameter of the tumor was ≥ 1.0 cm; 3) patients who had tumors with > 50% solid components. A total of 550 masses in 533 patients were excluded, including 256 masses without a history of primary tumors and without pathological confirmation, 82 masses with tumor size < 1 cm, 148 masses with a history of primary tumors, while without pathological confirmation or follow-up data, and 35 masses with ≤ 50% solid components. Finally, a total of 680 patients with 701 masses met the inclusion criteria, including 328 men and 352 women, who aged 18-90 years old, with a median age of 56 (47.75, 64.25) years old (**Figure 1**). There were 636 tumors confirmed by pathology, including 489 cases of adrenal adenoma, 58 cases of pheochromocytoma, 49 cases of metastases, 17 cases of lymphoma, 12 cases of ganglioneuroma, 6 cases of cortical carcinoma, and 5 cases of schwannoma. Besides, 65 cases were confirmed by clinical follow-up, all of which were metastases. Clinical follow-up confirmation refers to a history of the primary tumors, and a rapid enlargement of the mass within 6 months or a rapid shrinkage after chemotherapy (8).

### CT examination

All cases were scanned by plain CT. In medical centers A, a GE light speed 16-slice spiral CT or Optima540 16-slice spiral CT was used. The scanning pitch was 1.0 and 1.75 respectively, the layer thickness and layer distance of the former were 3.75mm, and the layer thickness and layer distance of the latter were 5mm. In medical center B, a GE optima 16-slice spiral CT was used, with scanning pitch of 1.75, layer thickness of 5mm and layer spacing of 5mm.

### Image assessment

**Figure 2:**
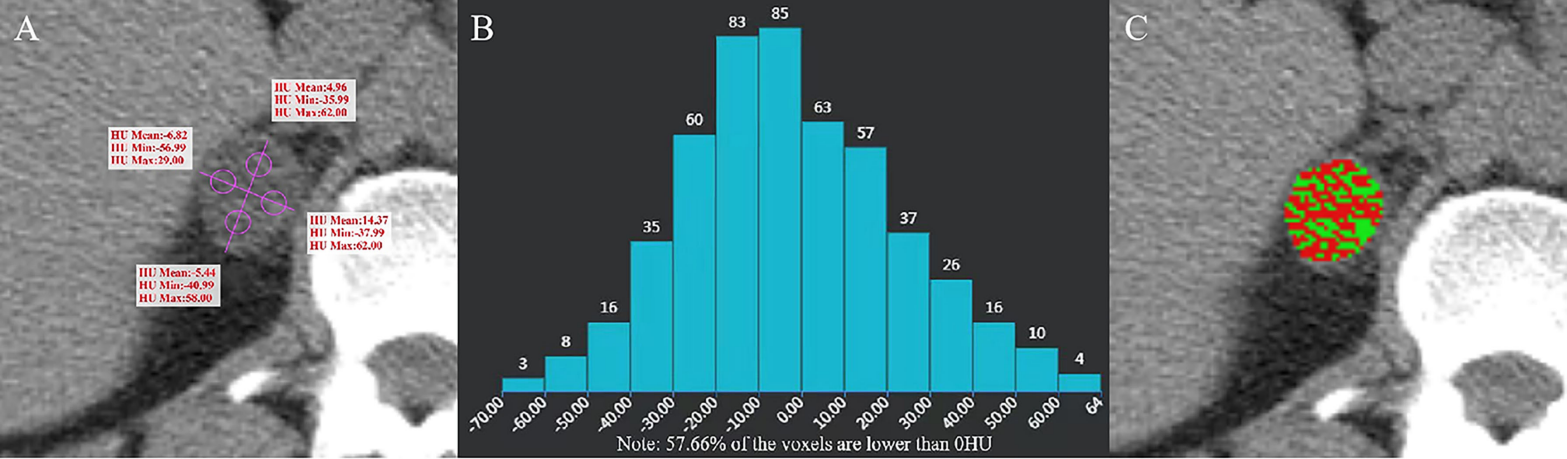
Adenoma of the right adrenal gland in a 47-year-old man. AVmean = 1.768 HU, AVmin = -42.99 HU **(A)**, and CTH negative pixels = 57.66% **(B)**. The CT value in the red area is ≤ 10 HU, and the CT value in the green area is > 10 HU **(C)**.

**Figure 3:**
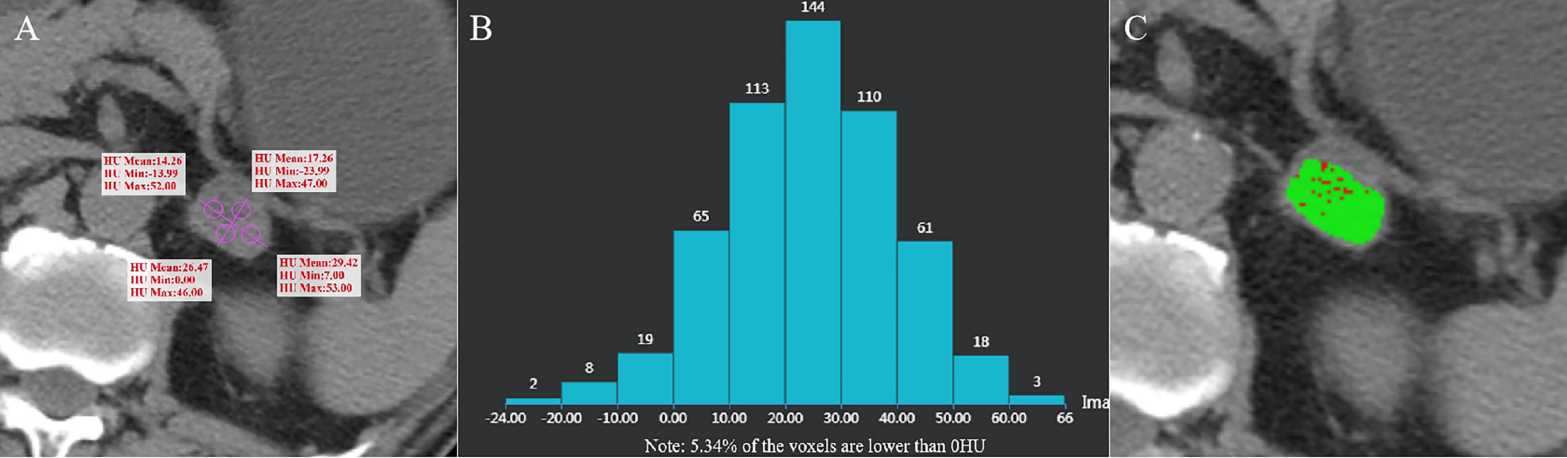
Adrenal adenoma of the left adrenal gland in a 62-year-old man. AV_mean_ =21.853 HU **(A)**, AV_min_ = -7.745 HU, and CTH negative pixel =5.34% **(B)**. The CT value in the red area is ≤ 10 HU, and the CT value in the green area is > 10 HU **(C)**.

**Figure 4:**
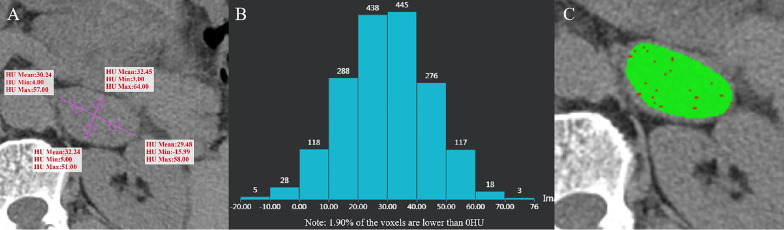
Ganglioneuroma of the left adrenal gland in a 55-year-old man. AV_mean_ = 31.103 HU, AV_min_ = -0.75 HU **(A)**, and CTH negative pixel =1.9% **(B)**. The CT value in the red area is ≤10 HU, and the CT value in the green area is > 10 HU **(C)**.

**Figure 5:**
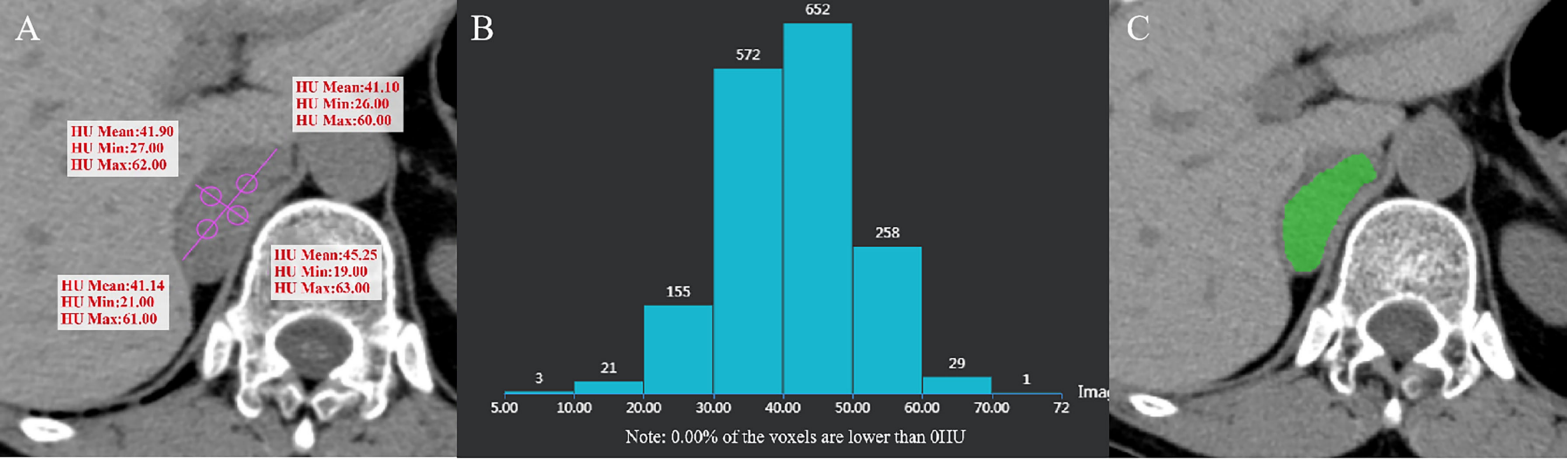
Pheochromocytoma of the right adrenal gland in a 64-year-old woman. AV_mean_ = 41.345 HU, AV_min_ = 23.25 HU **(A)**, and CTH negative pixel = 0 **(B)**. CT value in the green area is > 10 HU **(C)**.

The images were jointly analyzed by two radiologists who had worked for 8 and 6 years on the GE Advantage Windows 4.6 workstation (General Electric Inc., Boston, MA, USA). The largest slice of the tumor was selected for the measurement of CTH, and the circular or oval region of interest (ROI) was used as large as possible (7, 12). The histogram analysis software was used to depict the CT value distribution curve in ROI. The X-axis represents the distribution range of CT value in ROI, and the Y-axis represents the frequency of each CT value. The software automatically obtained the percentage of negative pixels in ROI. In the present study, AV_mean_ and AV_min_ were measured by a 4-point method, that is, the maximum transverse diameter and the longitudinal diameter were made on the maximum axial plane of the tumor to obtain the intersection point, the midpoint from the intersection point to the edge of the tumor was taken as the measurement point, and the ROI was 19 ~ 24 mm² (11) (**Figures 2**–**5**). When each ROI was selecting, it was attempted to avoid necrotic, hemorrhagic, and calcified areas. All measurements were made in triplicate, and the average value was calculated as the final value.

### Statistical analysis

Statistical analysis was performed using SPSS 25.0 software (IBM Corp., Armonk, NY, USA). The independent-samples t-test or the Mann-Whitney U test was used to compare AV_mean_, AV_min_, and CTH between adrenal adenomas and non-adenomas. AV_mean_ ≤ 10 HU, AV_min_ ≤ 0 HH, and CTH negative pixels ≥ 10% were analyzed by the Chi-squared test or the Fisher’s exact test. Receiver operating characteristic (ROC) curves of AV_mean_, AV_min_, and CTH in two medical centers were plotted to distinguish adrenal adenoma from non-adenoma, and value of area under the curve (AUC) was calculated. The sensitivity, specificity, and accuracy of AV_mean_ ≤ 10 HU, AV_min_ ≤ 0 HU, and CTH negative pixels ≥ 10% for diagnosing adrenal adenoma, and AV_min_ and CTH for adenoma with AV_mean_ > 10Hu were compared between the two medical centers. *P* value < 0.05 was considered statistically significant.

## Results

The distribution of gender, age and BMI in adenomas and non-adenomas was shown in **Table 1**. Among 489 cases of adrenal adenoma in the two medical centers, there were 297 cases of the left-sided adrenal adenoma and 192 cases of the right-sided adrenal adenoma, with a diameter of 10.0 ~ 51.2 mm (mean, 22.8 ± 8.2 mm) (**Figures 2**, **3**). Among 212 cases of non-adenoma, there were 110 cases of the left-sided adrenal adenoma and 112 cases of the right-sided adrenal adenoma, with a diameter of 10.0 ~ 68.5 mm (mean, 34.4 ± 13.9 mm) (**Figures 4**, **5**).

**Table 1:** Distribution of the three parameters in two centers.

Variable	Center A			Center B		
	Adenoma (n=403)	Non-adenoma (n=141)	P	Adenoma (n=86)	Non-adenoma (n=71)	P
Age	54.04 ± 12.52	59.30 ± 16.9	<0.001	53.54 ± 11.13	59.30 ± 10.90	0.004
Sex			<0.001			0.010
Male	155	93		36	45	
Female	248	48		50	26	
BMI	24.43 ± 3.63	21.68 ± 3.45	<0.001	24.38 ± 2.87	21.26 ± 3.03	<0.001
Diameter	22.63 ± 8.34	33.42 ± 14.25	<0.001	23.63 ± 8.11	34.68 ± 14.50	<0.001
Diagnosis method			<0.001			<0.001
Pathology	403	102		86	45	
Clinical follow-up	0	39		0	26	
AV_mean_, HU	9.02 ± 14.38	38.57 ± 6.96	<0.001	3.81 (-4.01, 17.00)	35.03 ± 6.44	<0.001
≤ 10, N (%)	238 (59.06)	0	<0.001	57 (66.27)	0	<0.001
> 10, N (%)	165 (40.94)	141 (100)		29 (33.73)	71 (100)	
AV_min_, HU	-16.76 (-30.75, -7)	12 (8.25, 21.38)	<0.001	-16.44 ± 16.93	16.58 ± 9.08	<0.001
≤ 0, N (%)	350 (86.85)	2 (1.42)	<0.001	71 (82.56)	2 (2.82)	<0.001
> 0, N (%)	53 (13.15)	139 (98.58)		15 (17.44)	69 (97.18)	
CTH, %	34.78 (10.00,58.82)	0 (0, 0.42)	<0.001	44.90 (6.96, 72.04)	0 (0, 0.36)	<0.001
Negative pixels ≥ 10%, N (%)	306 (75.93)	0	<0.001	58 (67.44)	0	<0.001
Negative pixels < 10%, N (%)	97 (24.07)	141 (100.00)		28 (32.56)	71 (100.00)	

AV_mean_: mean attenuation value; AV_min_: minimum attenuation value; CTH: CT histogram.

In center A and B, AV_mean_, AV_min_, and CTH of adenoma and non-adenoma were shown in **Table 1**, and these parameters were significantly lower among the adrenal adenomas (all *P* < 0.001). The proportions of AV_mean_ ≤ 10 HU, AV_min_ ≤ 0 HU, and CTH negative pixels ≥ 10% were also presented in **Table 1**, and these parameters have significant difference in the number of adenomas and non-adenomas (all *P* < 0.001). In center A, there were 2 cases of non-adenoma with AVmin ≤ 0 HU, including 1 case of lung cancer with adrenal metastasis and 1 case of ganglioneuroma (**Figure 4**). In center B, there were 2 cases of non-adenoma with minAVs ≤ 0 HU, all of which were ganglioneuroma.

In both the centers, showing AV_mean_ ≤ 10 HU, AV_min_ ≤ 0 HU, and CTH negative pixels ≥ 10% (all *P*<0.001), and In center A and B, the AUC of AV_mean_, AV_min_, CTH negative pixels for the differential diagnosis of adenoma and non-adenoma was 0.941~0.956 and 0.952~0.958, respectively. The corresponding sensitivity, specificity, and accuracy of AV_mean_ ≤ 10 HU, AV_min_ ≤ 0 HU, and CTH negative pixels≥10% were shown in **Table 2**.

**Table 2:** Comparison of diagnostic efficacy of three parameters for adenoma in two centers.

Variable		AUC	Sensitivity	Specificity	Accuracy
AV_mean_ ≤ 10	Center A	0.956	0.591	1.000	0.697
	Center B	0.956	0.663	1.000	0.815
AV_min_ ≤ 0	Center A	0.941	0.869	0.986	0.899
	Center B	0.958	0.826	0.972	0.892
CTH negative pixels ≥ 10%	Center A	0.948	0.759	1.000	0.822
	Center B	0.952	0.674	1.000	0.822

AV_mean_: mean attenuation value; AV_min_: minimum attenuation value; CTH: CT histogram.

Among adenoma with AV_mean_ >10 HU, the best threshold of AV_min_ in center A and center B were -0.250HU and 2.375HU, and the corresponding AUC, sensitivity and specificity were 0.858 and 0.846, 0.691 and 0.586, 0.986 and 0.958; the best threshold of CTH in center A and center B were 0.895% and 0.775%, and the corresponding AUC, sensitivity and specificity were 0.873 and 0.822, 0.818 and 0.724, 0.837 and 0.915 (**Table 3**).

**Table 3:** Comparison of diagnostic efficacy of AVmin and CTH for adenoma with AV_mean_ >10 HU in two centers.

Variable		AUC	Best threshold	Sensitivity	Specificity	Accuracy
AV_min_	Center A	0.858	-0.250	0.691	0.986	0.827
	Center B	0.846	2.375	0.586	0.958	0.850
CTH	Center A	0.873	0.895%	0.818	0.837	0.827
	Center B	0.822	0.775%	0.724	0.915	0.860

## Discussion

In the present study, we conducted a control study on cases with adrenal adenoma and non-adenoma from two medical centers using plain CT scan with AV_mean_, AV_min_, and CTH. The results showed that sensitivity and accuracy of AV_mean_ ≤ 10 HU were the lowest, those of AV_min_ ≤ 0 Hu were the highest, and those of CTH negative pixels ≥ 10% were in the middle. Moreover, specificity of AV_mean_ ≤ 10 HU and CTH negative pixels ≥ 10% was equal to 1.000, and that of AV_min_ ≤ 0 HU was slightly lower (0.972). The sensitivity, specificity, and accuracy of the AV_min_ ≤ 0 HU and CTH negative pixels ≥ 10% for the diagnosis of adrenal adenoma in center A were similar to those in center B. The specificity of AV_mean_ ≤ 10 HU in the diagnosis of adrenal adenoma in center A was consistent with that in center B, while the sensitivity was quite different (0.591 vs. 0.663), which could be related to the different inclusion criteria for surgical resection considered in the two medical centers. When AV_min_ in center A and center B was less than -0.250HU and 2.375HU, respectively, and CTH in center A and center B was greater than 0.895% and 0.775%, respectively, adenomas with AV_mean_ >10 HU could be effectively diagnosed, and AV_min_ had high specificity, CTH had high sensitivity.

It is noteworthy that AV_mean_, AV_min_, and CTH are closely correlated together. CTH was first proposed by Bae et al. for the differential diagnosis of adrenal masses (9). It can display all pixel values in ROI of tumors and record them in the form of histograms. The X-axis corresponds to the CT values, and the Y-axis represents the pixel frequency of each CT value. CTH can fully reflect the heterogeneity of pixels in different ROIs. The incidence of adenoma can be evaluated by the percentage of negative pixels in ROI, that is, the larger the percentage, the greater the incidence of adenoma. At present, the widely accepted threshold is CTH negative pixels ≥10%, with a sensitivity of 82.9-92.2% and a specificity of 98.2-100% for the diagnosis of adenoma (7–9). Moreover, AV_mean_ is the average value of all pixels in the ROI, which is equivalent to the average value of all pixels in CTH. The lower the negative pixel value and the larger the proportion in ROI, the greater the incidence of adenoma. The broadly accepted threshold is AV_mean_ ≤ 10 HU, with a sensitivity of 47.6-82.2% and a specificity of 93.3-100% for the diagnosis of adenoma (6–10). AV_mean_ is the minimum value of pixels in ROI, which is equivalent to the minimum value of pixels in CTH. The smaller the AV_mean_ and the larger distribution areas, the greater the incidence of adenoma. We previously used AV_mean_ ≤ 0 HU as the threshold for diagnosing adenoma, with a sensitivity of 90.1% and a specificity of 96.8% (11).

The premise of diagnosing adenoma with CTH negative pixels ≥ 10% and AV_mean_ ≤ 10 HU requires the ratio of negative pixels to total pixels in the ROI to reach a certain value. Therefore, when the number of negative pixels in the tumor is small and the distribution is sparse, and the ratio of negative pixels to total pixels is lower than this value, the above-mentioned two criteria will be difficult to diagnose adrenal adenoma, especially for AV_mean_ ≤ 10 HU. However, AV_min_ ≤ 0 HU is different, because even if the negative pixels in the tumor are limited and sparse, as long as the ROI contains negative pixels, it can be judged as an adrenal adenoma. The present study showed that using AV_min_ and CTH was effective in differentiating adenoma with AV_mean_ > 10 HU, the former with high specificity and the latter with high sensitivity. Besides, using AV_min_ ≤ 0 HU as the best threshold to diagnose adenoma, 44 cases (10.92%) and 13 cases (18.31%) of adenoma with CTH negative pixels < 10% could be diagnosed in center A and center B. It should be noted that 4 cases (1.89%) with AV_min_ ≤ 0 HU were non-adenoma in the two centers, which may be related to sparse visible components (e.g., nucleus) in the stroma of ganglioneuroma, edema in some cases, and small necrotic areas in the measurement of metastatic tumors that could not be distinguished by naked eyes.

Park et al. reported different results due to different measurement locations and ROIs (13). At present, in the selection of ROI for AV_mean_ and CTH, scholars mainly use circular or oval shape, and the measured area is 1/2-2/3 of the largest layer (3, 6, 9), or take the largest area as far as possible (1, 2, 7, 8, 12). There are some shortcomings in this measurement method. Firstly, the definition of measurement area is extremely wide. When measurement is carried out on the largest cross-sectional area of the tumor, from more than 1/2 to cover the whole cross-sectional area as much as possible, several measurement methods can be used, which are corresponded to different measured areas, and different measured areas may cause certain differences. Secondly, it is difficult to avoid necrosis (especially in the central area of tumor that is prone to necrosis), hemorrhage, calcification, etc., when measuring the area of the largest slice of more than 1/2 tumor. Necrosis may increase the percentage of negative pixels, resulting in an increase in the false positive diagnostic rate of AV_mean_ and CTH, while hemorrhage and calcification may increase the percentage of positive pixels, that is, AV_mean_ ≥ 10 HU and/or CTH negative pixels < 10%, resulting in difficulties in the diagnosis. In order to avoid these shortcomings, the present study improved our previous measurement method (11), changing 5 points to 4 points, that is, eliminating the measurement points in the central area of the tumor. This newly proposed measurement method have the following advantages: 1) the measurement method is clearly defined, and it is highly repeatable; 2) the central area of the tumor which is prone to necrosis can be avoided; 3) multi-point measurement can reflect the heterogeneity of different points in tumor; 4) calculation of average value of four measurement points can reduce the measurement error.

There were four deficiencies in the present study. Firstly, 65 cases of adrenal metastasis were clinically confirmed rather than the pathology. However, the majority of the patients who were diagnosed with adrenal metastasis have lost the opportunity of surgery, and it was no longer necessary to perform invasive surgery or puncture, thus, we strictly adopted the clinical criteria for judging metastatic tumors proposed by Halefoglu (8). Secondly, adrenal washout CT and chemical shift MRI are of great significance in the differential diagnosis of adrenal masses, especially when plain CT scan is not predominant in the diagnosis of for lipid-poor adenomas and non-adenomatous lesions (14–16). However, this study aimed to explore the clinical value of three measurement methods in plain CT scan. In the future studies, we will combine these three measurement methods with washout CT and chemical shift MRI for a more comprehensive analysis. Thirdly, the study aimed to compare the differential diagnostic efficacy of three CT parameters for adrenal tumors. Functional diagnosis (cortisol or aldosterone assessment) of the masses has not been analyzed, and comprehensive analysis combined with these laboratory parameters will be our future direction. Finally, there might be selection bias due to the retrospective analysis of the data. Hence, additional prospective multicenter controlled studies are required to eliminate the above-mentioned limitations.

In conclusion, AV_mean_ ≤ 10 HU, AV_min_ ≤ 0 HU, and CTH negative pixels ≥10% have high diagnostic efficiency for adrenal adenomas and high consistency between the two medical centers. Even for adenomas with AV_mean_> 10 HU, AV_min_ and CTH could still distinguish them to a great extent. The complementarity of these three parameters could enhance the role of plain CT scan in the differential diagnosis of adrenal adenoma, and assist clinicians to develop personalized treatments.

## Data availability statement

The original contributions presented in the study are included in the article/Supplementary Material. Further inquiries can be directed to the corresponding authors.

## Ethics statement

The studies involving human participants were reviewed and approved by Affiliated Hangzhou First People’s Hospital, Zhejiang University School of Medicine, The Quzhou Affiliated Hospital of Wenzhou Medical University, and Quzhou People’s Hospital. The ethics committee waived the requirement of written informed consent for participation.

## Author contributions

ZD, MZ, and ZH conceived and designed the study. ZH, MW, and XZ collected the clinical and image data of all cases. ZH, MW, and PW reviewed, analyzed, and classified the imaging data. PW and HZ performed statistical analysis. ZH and MW wrote the first draft of the manuscript. All authors contributed to manuscript revision and approved the submitted version.

## Funding

This study was supported by the National Natural Science Foundation of China (81871337), the Medical Science Research Program of Zhejiang Province (2020RC091, 2021RC024), the Key Laboratory of Clinical Cancer Pharmacology and Toxicology Research of Zhejiang Province (2020E10021).

## Conflict of interest

The authors declare that the research was conducted in the absence of any commercial or financial relationships that could be construed as a potential conflict of interest.

## Publisher’s note

All claims expressed in this article are solely those of the authors and do not necessarily represent those of their affiliated organizations, or those of the publisher, the editors and the reviewers. Any product that may be evaluated in this article, or claim that may be made by its manufacturer, is not guaranteed or endorsed by the publisher.
